# Supplementary material for: Primary TSC2-/meth Cells Induce Follicular Neogenesis in an Innovative TSC Mouse Model
Source: Int J Mol Sci. 2022 Aug 26;23(17):9713. doi: 10.3390/ijms23179713 (PMC9456283; doi:10.3390/ijms23179713)
Supplement: Supplementary file 1 [file ijms-23-09713-s001.zip › ijms-1832000-supplementary.pdf]

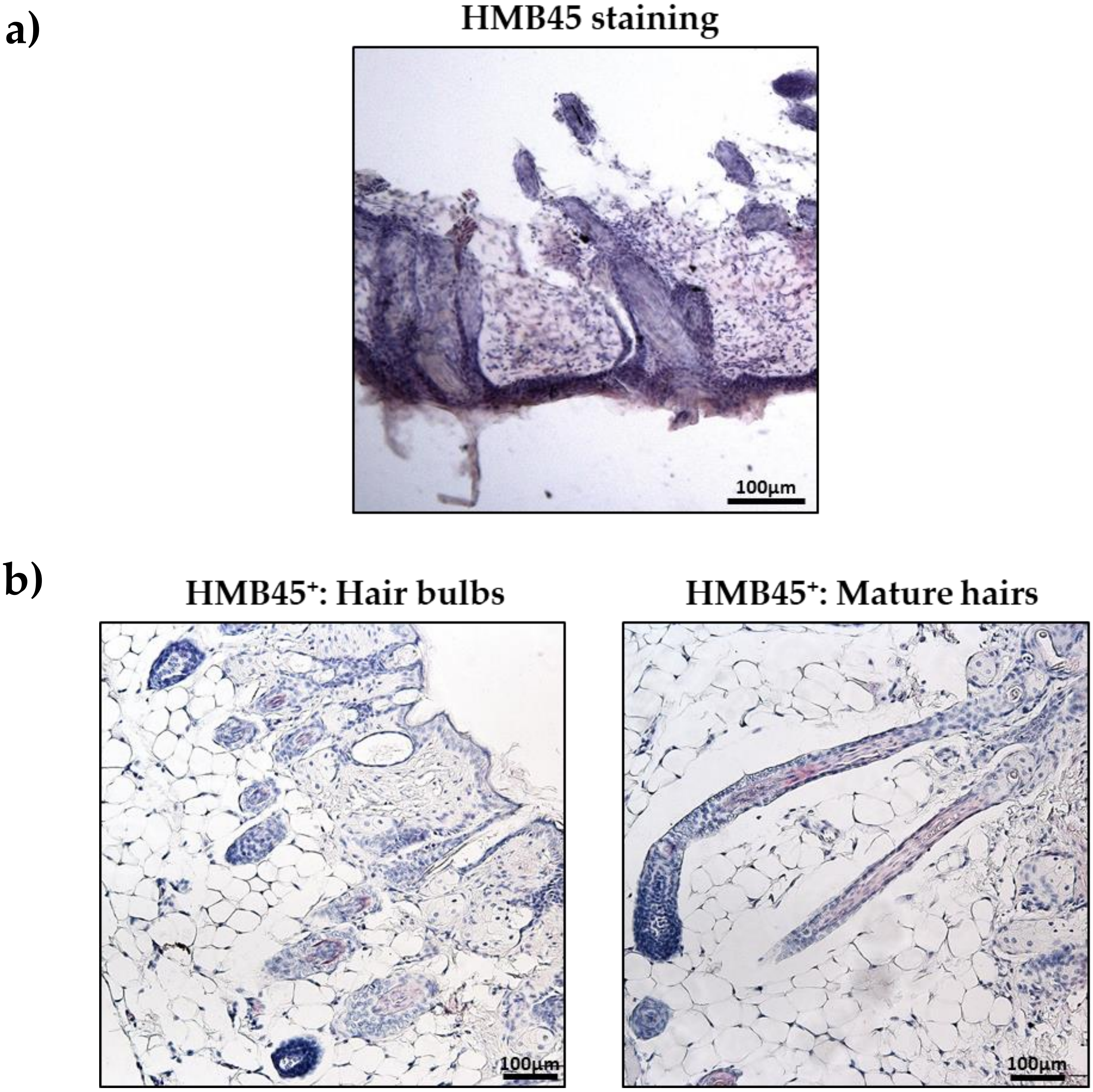

Supplemental Figure S1: (a) Low magnification of the images in Figure 2a showing the HMB45 antibody-immunohistochemistry of skin section from control mice. **(b)** Low magnification of the images in Figure 2b showing the immunohistochemistry with HMB45 antibody of skin section of mice injected with $TSC2^{-/meth}$ cells. HMB45-positive cells are stained in red (highlighted by arrows). Nuclei are stained with haematoxylin. Scale bar: 100µm

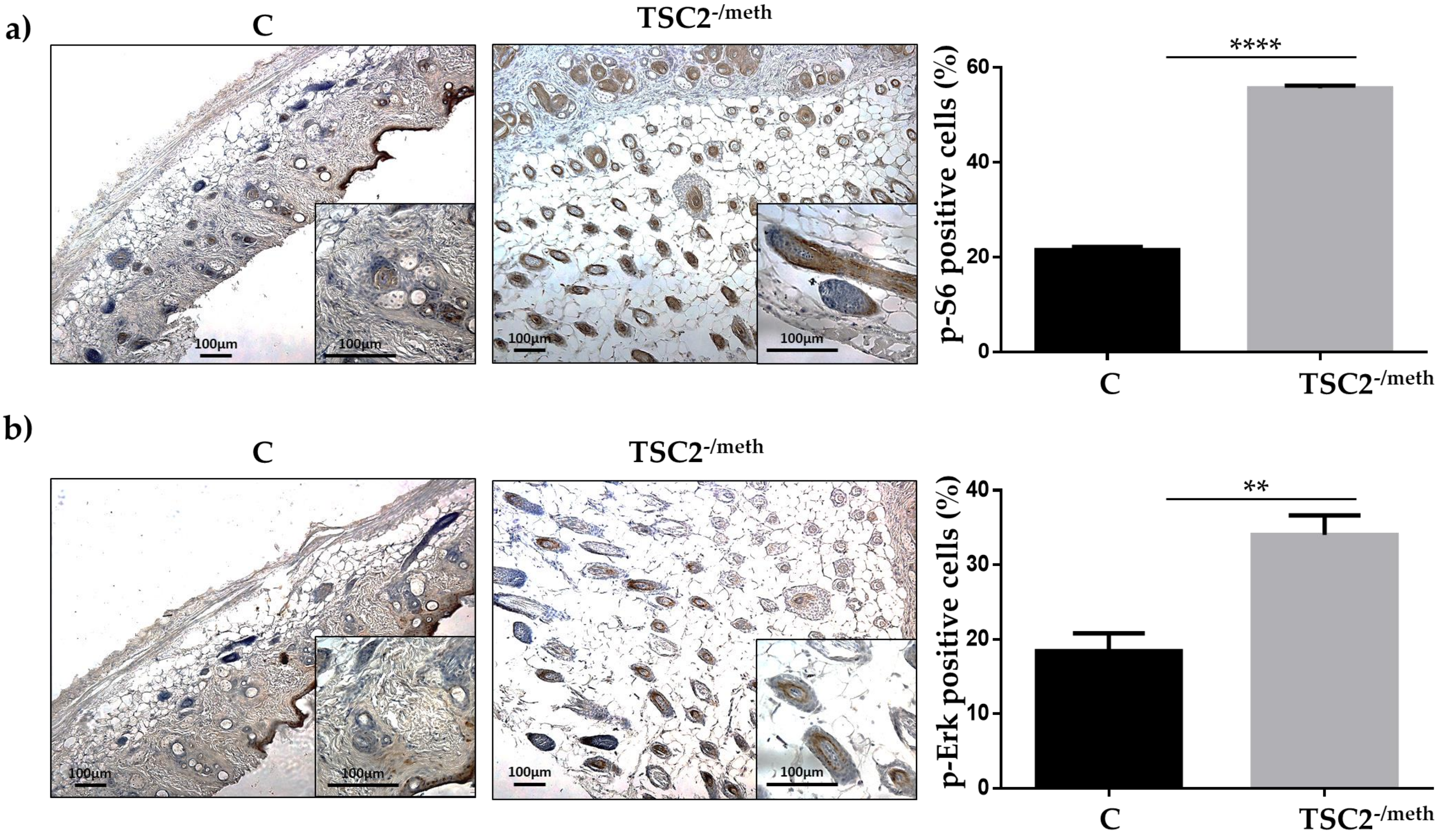

Supplemental Figure S2: Representative immunohistochemistry for phospho-S6 **(a)** and phospho-Erk **(b)** positivity in the skin of control mice (C) and mice injected with tuberin-null cells ($TSC2^{-/meth}$). For the images of skin from mice injected with $TSC2^{-/meth}$ cells, they are low and high magnification of Figure 3a and 3b, respectively. Scale bar: 100 µm. The histograms represent the percentage of phospho-S6 or phospho-Erk positive cells in the skin section of mice injected with $TSC2^{-/meth}$ cells compared to controls. N=3, Student's *t* Test, ****$p<0.0001$; ** $p<0.01$

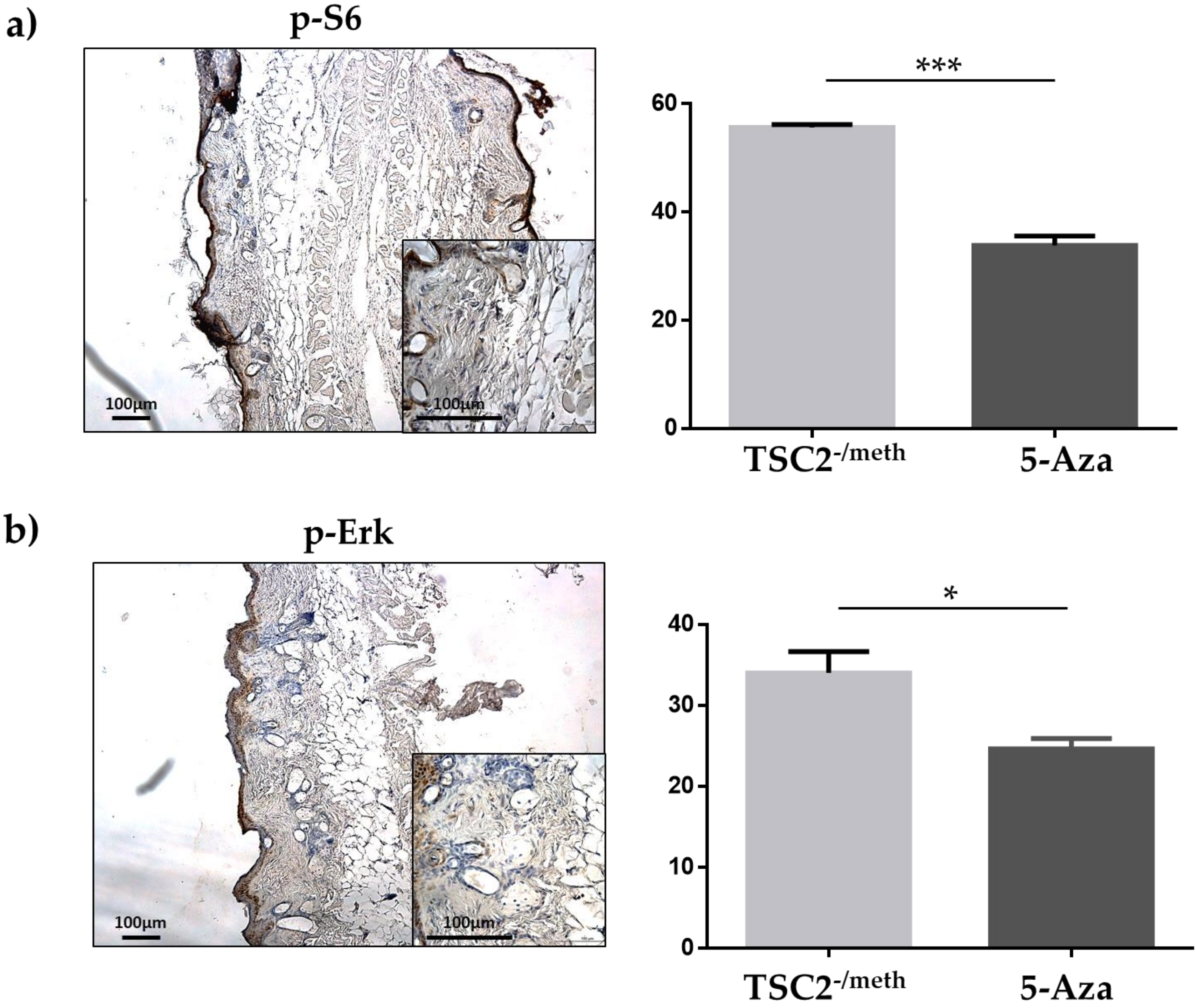

Supplemental Figure S3: Representative immunohistochemistry for phospho-S6 **(a)** and phospho-Erk **(b)** positivity in the skin of mice injected with tuberin-null cells and treated with 5-Azacytidine (5-Aza) for 4 days. **(a)** and **(b)** images are the low and high magnification of the Figure 4a and b, respectively. Scale bar: 100 μm. The histograms represent the percentage of phospho-S6 or phospho-Erk positive cells in the skin section of mice injected with TSC2$^{-/meth}$ cells and treated with 5-Aza compared to mice injected with TSC2$^{-/meth}$ cells. N=3, Student's *t* Test, *p<0.05; *** p<0.001
